# A negative correlation of low estimated glucose disposal rate with significant liver fibrosis in adults with NAFLD and obesity: results from NHANES 2017–2020

**DOI:** 10.1186/s12876-025-03798-y

**Published:** 2025-03-29

**Authors:** Xue-Cheng Tong, Kai Liu, Jun-Ying Xu, Xiu-Jun Zhang, Yuan Xue

**Affiliations:** 1 Department of Infectious Diseases, Changzhou Third People’s Hospital, Changzhou, China; 2 Department of Infectious Diseases, Changzhou Clinical College of Xuzhou Medical University, Changzhou, China; 3 Department of Pharmacy, Changzhou Clinical College of Xuzhou Medical University, Changzhou, China; 4 Department of Liver Diseases, Changzhou Third People’s Hospital, Changzhou Medical Center, Nanjing Medical University, No. 300 Lanling North Road, Changzhou, Jiangsu 213000 China

**Keywords:** Body mass index, Controlled Attenuation parameter, Estimated glucose disposal rate, Liver fibrosis, Nonalcoholic fatty liver disease

## Abstract

**Background and aim:**

Nonalcoholic fatty liver disease (NAFLD) with liver fibrosis is associated with liver-related mortality and cardiovascular disease. Estimated glucose disposal rate (eGDR), which is an insulin resistance–related index, is related to the mortality caused by NAFLD. This study aimed to investigate the predictive value of eGDR for liver fibrosis in patients with NAFLD.

**Methods:**

The data from the National Health and Nutrition Examination Survey 2017–2020.03 were analyzed in the present study. NAFLD was diagnosed using the controlled attenuation parameter (CAP) tests using FibroScan^®^ model 502 V2 Touch.

**Results:**

The data from 1585 individuals were analyzed, including 224 with significant fibrosis and 1361 with nonsignificant fibrosis. Individuals with significant fibrosis were older and had higher CAP values and lower eGDRs (both *P* < 0.01). A negative correlation was found between eGDR and stiffness degrees (odds ratio: 0.643, 95% confidence interval: 0.643–0.726, *P* < 0.001); the correlation was also significant after adjusting for age, sex, and ethics (*P* < 0.001). For participants with obesity and overweight, eGDR was negatively correlated with age, CAP, body mass index (BMI), waist circumference, C-reactive protein level, and white blood cell (WBC) count (all *P* < 0.05). The multivariate analysis revealed that age, eGDR, BMI, aspartate aminotransferase (AST), and WBC and platelet (PLT) counts (all *P* < 0.05) were independent risk factors for significant fibrosis. A model incorporating eGDR, BMI, age, AST, WBC, and PLT had an AUROC of 0.822, and was superior to conventional noninvasive scoring systems, including the AST-to-PLT ratio index, fibrosis-4 level, and gamma-glutamyl transpeptidase to platelet ratio for individuals with obesity (all *P* < 0.01).

**Conclusion:**

Low eGDR was negatively correlated with liver fibrosis in individuals with NAFLD and obesity, and a model incorporating eGDR, BMI, age, AST, WBC, and PLT demonstrated strong predictive value for fibrosis evaluation.

## Introduction

Nonalcoholic fatty liver disease (NAFLD) is characterized by the presence of steatosis in > 5% of hepatocytes in patients without a history of heavy drinking (daily alcohol intake of > 20 g for women or 30 g for men) and without other identified etiology of steatosis. Patients with NAFLD can develop progressive liver fibrosis, cirrhosis, hepatocellular carcinoma, and cardiovascular disease [1]. NAFLD has emerged as a global health challenge, with its remarkably high prevalence of 30.2% and its association with serious health consequences [2–4].

NAFLD with advanced liver fibrosis is associated with liver-related mortality and cardiovascular disease [5]. Hence, identifying patients with significant liver fibrosis and at high risk of disease progression is crucial. Although liver biopsy is efficient, it is invasive and unfeasible for most patients. Noninvasive scoring systems (NSSs), including aminotransferase-to-platelet ratio index (APRI), fibrosis-4 (FIB-4) level, and gamma-glutamyl transpeptidase to platelet ratio (GPR), can accurately evaluate liver fibrosis in patients with chronic viral hepatitis. However, the prediction value is not optimal in patients with NAFLD [6, 7]. The sensitivity and specificity of FIB-4 are 30.4% and 54.8% in diagnosing metabolic dysfunction-associated steatotic liver disease [6]. More convenient and accurate methods are needed to monitor fibrosis progression in clinical practice.

Obesity is a significant risk factor for metabolic disorders, with visceral adipose tissue affecting insulin sensitivity and aggravating disease progression [8]. The prevalence of NAFLD demonstrates a marked increase among overweight and obese individuals compared to those with normal-weight [9, 10]. Notably, metabolic dysfunction-associated steatohepatitis exhibits particularly high incidence rates in populations with severe obesity [11]. However, the clinical management of these conditions faces challenges due to the lack of reliable non-invasive biomarkers for predicting steatohepatitis [11]. Despite these limitations, body mass index (BMI) remains the predominant clinical parameter for classifying individuals into overweight and obesity categories.

Increasing evidence supports the association between insulin resistance (IR) and NAFLD in patients with and without obesity [12]. IR was also associated with liver fibrosis in nondiabetic patients with NAFLD [13]. IR-related indexes can serve as biomarkers to predict outcomes or as surrogate targets to mitigate liver damage progression [14, 15]. The estimated glucose disposal rate (eGDR), serving as a reliable indicator of IR, is calculated through a formula that incorporates three key parameters: waist circumference (WC), hypertension status, and hemoglobin A1c (HbA1c) levels [16]. Among these parameters, WC represents a well-established measure of central adiposity, whereas both hypertension and HbA1c serve as important markers of metabolic dysregulation. Lower eGDR was reported to be associated with high mortality caused by NAFLD [15]. Recent study has indicated that individuals with severe obesity might be at higher risk of advanced liver fibrosis [11]. Given that screening for significant fibrosis is advised in patients with NAFLD, and eGDR is an important IR-related index, it is important to explore the relationship between eGDR and fibrosis degrees in NAFLD patients, especially regarding its variation with BMI.

This study investigated the effectiveness of eGDR in predicting fibrosis degrees in US adults with obesity, overweight, and normal weight, and developed a new optimal model for fibrosis evaluation.

## Materials and methods

### Data sources

The data from the National Health and Nutrition Examination Survey (NHANES) 2017–2020.03 were analyzed in the present study. Data on demographic characteristics, physical examination, laboratory tests, and questionnaire were collected.

**Fig. 1:**
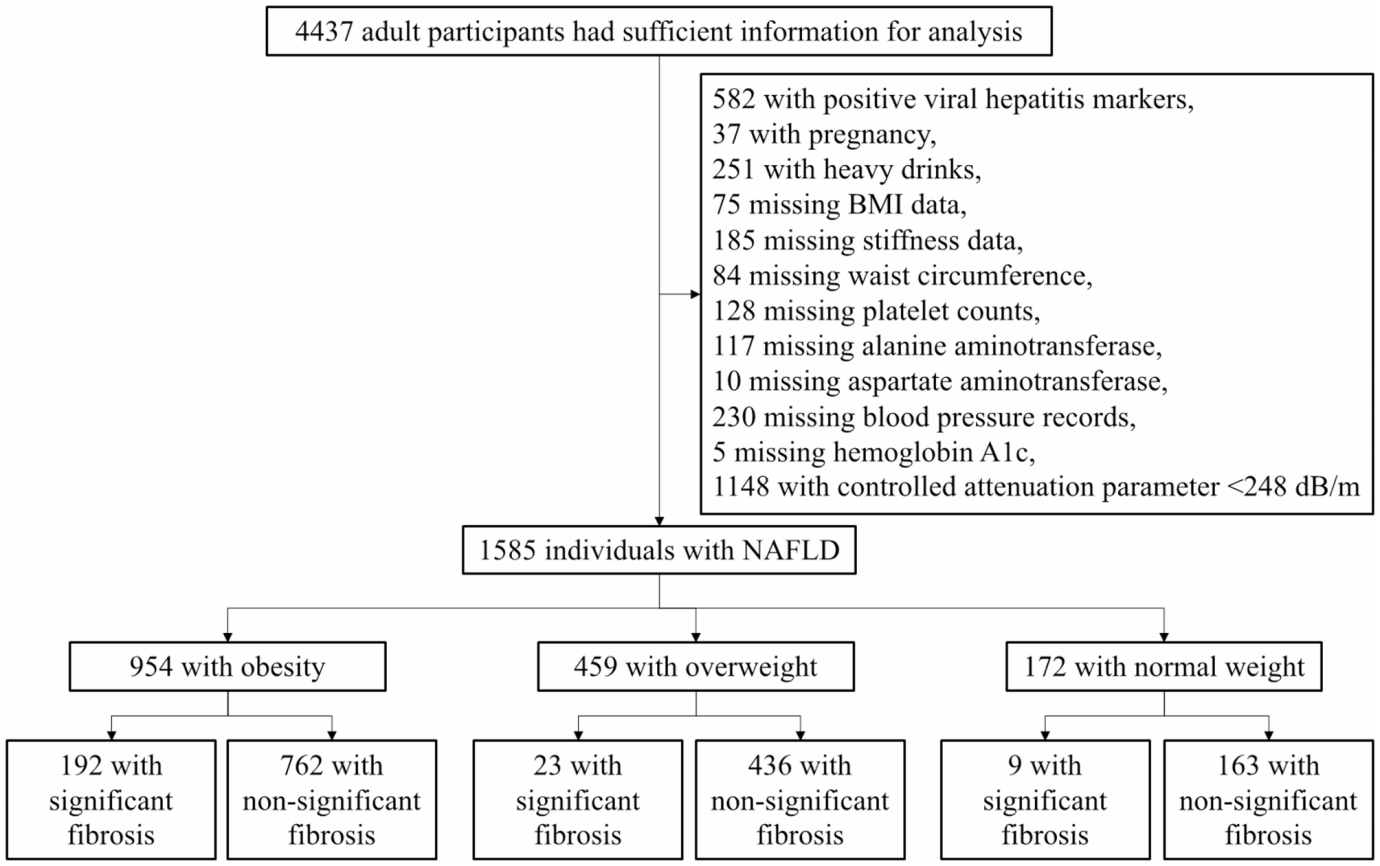
Flow chart of the study

The data were pooled using EmpowerStats (www.empowerstats.com) according to the respondent sequence number. A total of 4437 adult participants underwent tests for glycohemoglobin, viral hepatitis, high-sensitivity C-reactive protein (CRP), plasma fasting glucose, standard biochemistry profile, urine pregnancy tests, total cholesterol (TC), triglyceride (TG), and complete blood counts. Also, they were subjected to FibroScan model 502 V2 Touch tests and participated in a survey for alcohol use, blood pressure, and diabetes. As shown in the flow chart (Fig. 1), the following individuals were excluded: 582 with positive viral hepatitis markers, 37 who were pregnant, 251 heavy drinkers, 75 missing BMI data, 185 missing stiffness data, 84 missing WC, 128 missing platelet counts, 127 missing aspartate aminotransferase (AST) and alanine aminotransferase (ALT) data, 230 missing blood pressure records, 5 missing HbA1c, and 1148 with a controlled attenuation parameter (CAP) < 248 dB/m. Men who had ≥ 3 drinks per day or women who had ≥ 2 drinks per day were defined as heavy drinkers [17, 18]. In accordance with established diagnostic criteria outlined in previous publications, participants exhibiting CAP < 248 dB/m were excluded from the analysis. Consequently, a total of 1,585 individuals meeting the diagnostic threshold of CAP ≥ 248 dB/m were identified as having NAFLD [19, 20]. Significant fibrosis was considered when liver stiffness was ≥ 8.1 kPa [21].

Apart from AST and ALT, data on covariates, including γ-glutamyl transferase (GGT), uric acid (UA), CRP, TC, and TG levels, as well as WBC and PLT counts, were also collected.

### Definitions of eGDR and significant fibrosis

The eGDR was calculated using the following formula: eGDR = 21.158 − (0.09 × WC) − (3.407 × HT) − (0.551 × HbA1c) [WC = waist circumference (cm), HT = hypertension (yes = 1/no = 0), and HbA1c = HbA1c (%)].The participants were divided into three groups: normal weight (BMI < 25 kg/m^2^), overweight (BMI 25–29.9 kg/m^2^), and obesity (BMI ≥ 30 kg/m^2^), calculated as BMI = body mass (kg)/height^2^ (m^2^). Conventional NSSs, including FIB-4, GPR, and APRI, were calculated as previously reported [22].

### Statistical analysis

The data were analyzed using SPSS version 25.0 (NY, USA) and MedCalc version 20.1.0 (Medcalc Software, Mariakerke, Belgium). The continuous variables were expressed as median with interquartile range, and the categorical values as frequencies. The Mann–Whitney *U* test was used for continuous variables, whereas the chi-square test was used for categorical variables. Multivariate logistic regression analysis was performed to analyze independent risk factors for significant fibrosis. A website tool (https://hiplot.com.cn) was used to create the correlation heatmaps. Several NSSs were compared according to the area under the curve of the receiver operating characteristics. A two-sided *P* value < 0.05 indicated a statistically significant difference.

## Results

### Characteristics of participants

The data from 1585 individuals were analyzed, including 224 participants with significant fibrosis and 1361 with nonsignificant fibrosis (Table 1). Individuals with significant fibrosis were older and had higher levels of various biomarkers, including CAP, BMI, ALT, AST, GGT, UA, CRP, and WBC count (all *P* < 0.01) and lower eGDR and platelet count (all *P* < 0.01). More participants in the significant fibrosis group had hypertension (*P* = 0.039) and diabetes (*P* < 0.01).

**Table 1:** Characteristics of participants with NAFLD and significant fibrosis

Variable	Nonsignificant fibrosis (*n* = 1361)	Significant fibrosis(n=224)	*P* value (	
Age	53.00 (18.00–80.00)	57.00 (18.00–80.00)	0.004	
Stiffness, kPa	5.00 (1.60–8.10)	10.75 (8.20–69.10)	< 0.001	
CAP, dB/m	294.00 (248.00–400.00)	342.00 (251.00–400.00)	< 0.001	
eGDR	8.09 (1.45–12.42)	5.42 (1.46–11.22)	< 0.001	
BMI, kg/m^2^	30.80 (16.30–65.30)	37.35 (19.70–67.00)	< 0.001	
ALT, U/L	19.00 (3.00–159.00)	24.00 (10.00–181.00)	< 0.001	
AST, U/L	19.00 (8.00–112.00)	21.00 (9.00–272.00)	< 0.001	
GGT, U/L	23.00 (5.00–300.00)	30.00 (10.00–708.00)	< 0.001	
UA, mmol/L	327.10 (95.20–660.20)	362.80 (178.40–612.60)	< 0.001	
CRP, mg/L	2.48 (0.11–124.34)	3.87 (0.20–49.40)	0.001	
TC, mmol/L	4.72 (1.97–9.26)	4.45 (1.99–11.07)	< 0.001	
TG, mmol/L	1.20 (0.20–8.81)	1.32 (0.24–15.89)	0.042	
WBC, ×10^9^/L	6.70 (1.90–24.00)	7.40 (3.60–20.60)	< 0.001	
Platelet, ×10^9^/L 245.00(71.00—662.00)		231.00(72.00—540.00)	0.007	
Sex			0.308	
*Male*	673 (49.45%)	119 (53.12%)		
*Female*	688 (50.55%)	105 (46.88%)		
Ethnicity			0.475	
*Mexican American*	205 (15.06%)	34 (15.18%)		
*Other Hispanic*	149 (10.95%)	25 (11.16%)		
*Non-Hispanic White*	490 (36.00%)	92 (41.07%)		
*Non-Hispanic Black*	299 (21.97%)	48 (21.43%)		
*Non-Hispanic Asian*	142 (10.43%)	15 (6.70%)		
*Other Race*	76 (5.58%)	10 (4.46%)		
Hypertension	309 (22.70%)	65 (29.02%)	0.039	
Diabetes	222(16.3%)	90(40.2%)	< 0.001	

The data were expressed as median (IQR) for continuous variables and *n* (%) for categorical values, and compared using the Mann–Whitney *U* test or chi-square test. ALT, Alanine aminotransferase; AST, aspartate transaminase; BMI, body mass index; CAP, controlled attenuated parameter; CRP, C-reactive protein; eGDR, estimated glucose disposal rate; GGT, γ-glutamyl transferase; NAFLD, nonalcoholic fatty liver disease; TC, total cholesterol; TG, triglyceride; UA, uric acid; WBC, white blood cell

### Association between eGDR and liver stiffness

A negative correlation was found between eGDR and stiffness degrees [odds ratio (OR): 0.643, 95% confidence interval (CI): 0.643–0.726), *P* < 0.001], and this correlation was also significant after adjusting for age, sex, and ethics (OR: 0.668, 95% CI: 0.626–0.713, *P* < 0.001). After adjusting for variables such as age, sex, ethics, CAP, BMI, platelet, ALT, AST, GGT, TC, TG, CRP, and UA levels, and WBC count, eGDR remained negatively correlated with stiffness degrees (OR: 0.840, 95% CI: 0.769–0.917, *P* < 0.001).

**Fig. 2:**
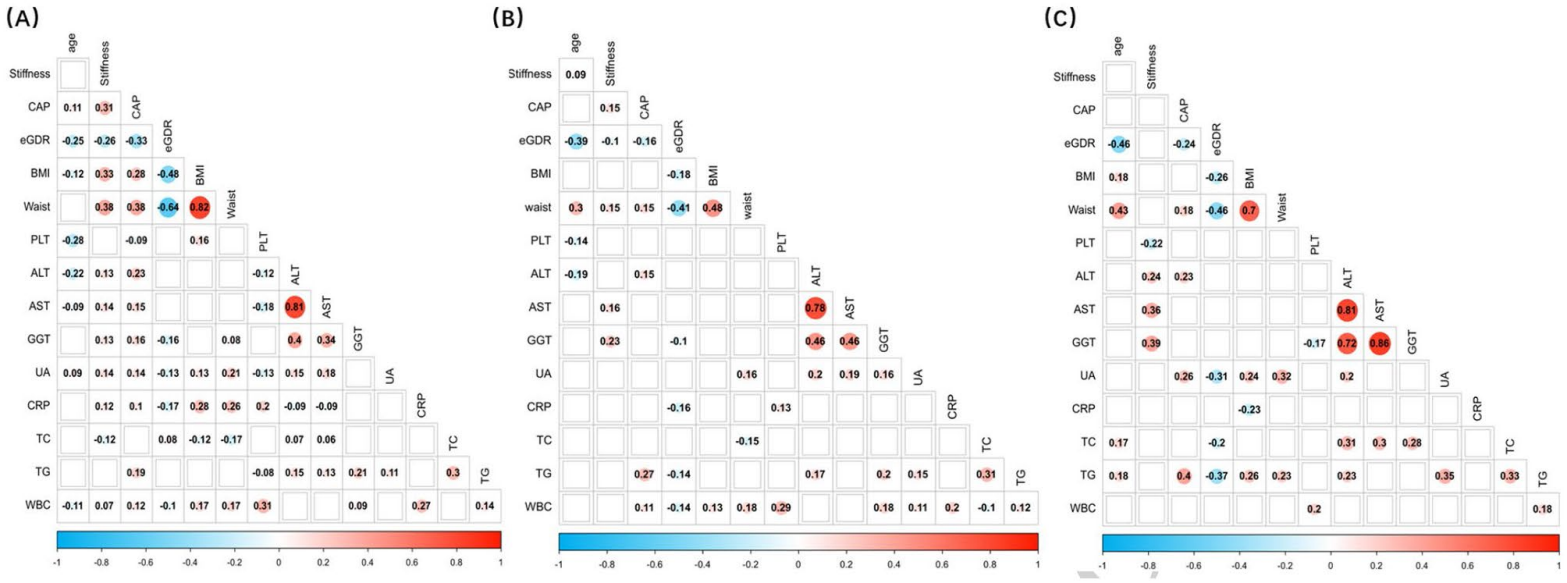
Correlation of eGDR with clinical parameters in individuals with NAFLD and obesity (**A**), overweight (**B**), and normal weight (**C**). ALT, Alanine aminotransferase; AST, aspartate transaminase; BMI, body mass index; CAP, controlled attenuated parameter; CRP, C-reactive protein; eGDR, estimated glucose disposal rate; GGT, γ-glutamyl transferase; NAFLD, nonalcoholic fatty liver disease; PLT, platelet; TC, total cholesterol; TG, triglyceride; UA, uric acid; WBC, white blood cell

Furthermore, the participants were divided into three groups: obesity (*n* = 954), overweight (*n* = 459), and normal-weight (*n* = 172). In participants with obesity and overweight, eGDR was negatively correlated with age, CAP, BMI, WC, CRP level, WBC count, and liver stiffness (all *P* < 0.05). For participants with normal weight, negative correlations were observed between eGDR and age, CAP, BMI, WC, TC level, and TG level (all *P* < 0.05). However, the association between eGDR and liver stiffness was not significant (*r*^2^ = − 0k.077, *P* = 0.314) (Fig. 2).

### Development of a new model for predicting significant fibrosis

As shown in Table 2, the univariate analysis showed that age, eGDR, BMI, ALT and AST levels, and WBC and platelet counts were associated with significant fibrosis (all *P* < 0.01). Then, the multivariate analysis showed that age (*P* < 0.001), eGDR (*P* < 0.001), BMI (*P* < 0.001), AST level (*P* < 0.001), and WBC (*P* = 0.025) and platelet (*P* = 0.002) counts were independent risk factors for significant fibrosis.

**Table 2:** Risk factors for significant fibrosis in participants with NAFLD

Variable	Univariate			Multivariate		
	Odds ratio	95% CI	*P*	Odds ratio	95% CI	*P*
Age	1.013	1.004–1.022	0.004	1.022	1.010–1.034	< 0.001
Sex	1.159	0.873–1.538	0.308			
eGDR	0.683	0.643–0.726	< 0.001	0.815	0.752–0.883	< 0.001
BMI	1.126	1.104–1.148	< 0.001	1.111	1.083–1.140	< 0.001
ALT	1.024	1.016–1.032	< 0.001			
AST	1.041	1.027–1.055	< 0.001	1.050	1.034–1.066	< 0.001
WBC	1.138	1.069–1.213	< 0.001	1.091	1.011–1.177	0.025
Platelet	0.997	0.995–0.999	0.008	0.996	0.993–0.998	0.002

ALT, Alanine aminotransferase; AST, aspartate transaminase; BMI, body mass index; eGDR, estimated glucose disposal rate; NAFLD, nonalcoholic fatty liver disease; TC, total cholesterol; WBC, white blood cell

A new model was developed based on the multivariate analysis: Model(eGDR_BMI_age_AST_WBC_PLT)(y = 1)

$$\:\frac{\begin{array}{l}{\text{e}\text{x}\text{p}(-5.976-0.203\text{*}\text{e}\text{G}\text{D}\text{R}+0.106\text{*}\text{B}\text{M}\text{I}}\\{+0.022\text{*}\text{a}\text{g}\text{e}+0.049\text{*}\text{A}\text{S}\text{T}+0.086\text{*}\text{W}\text{B}\text{C}-0.004\text{*}\text{P}\text{L}\text{T})}\end{array}}{\begin{array}{l}{1+\text{e}\text{x}\text{p}(-5.976-0.203\text{*}\text{e}\text{G}\text{D}\text{R}+0.106\text{*}\text{B}\text{M}\text{I}}\\{+0.022\text{*}\text{a}\text{g}\text{e}+0.049\text{*}\text{A}\text{S}\text{T}+0.086\text{*}\text{W}\text{B}\text{C}-0.004\text{*}\text{P}\text{L}\text{T})}\end{array}}$$

**Fig. 3:**
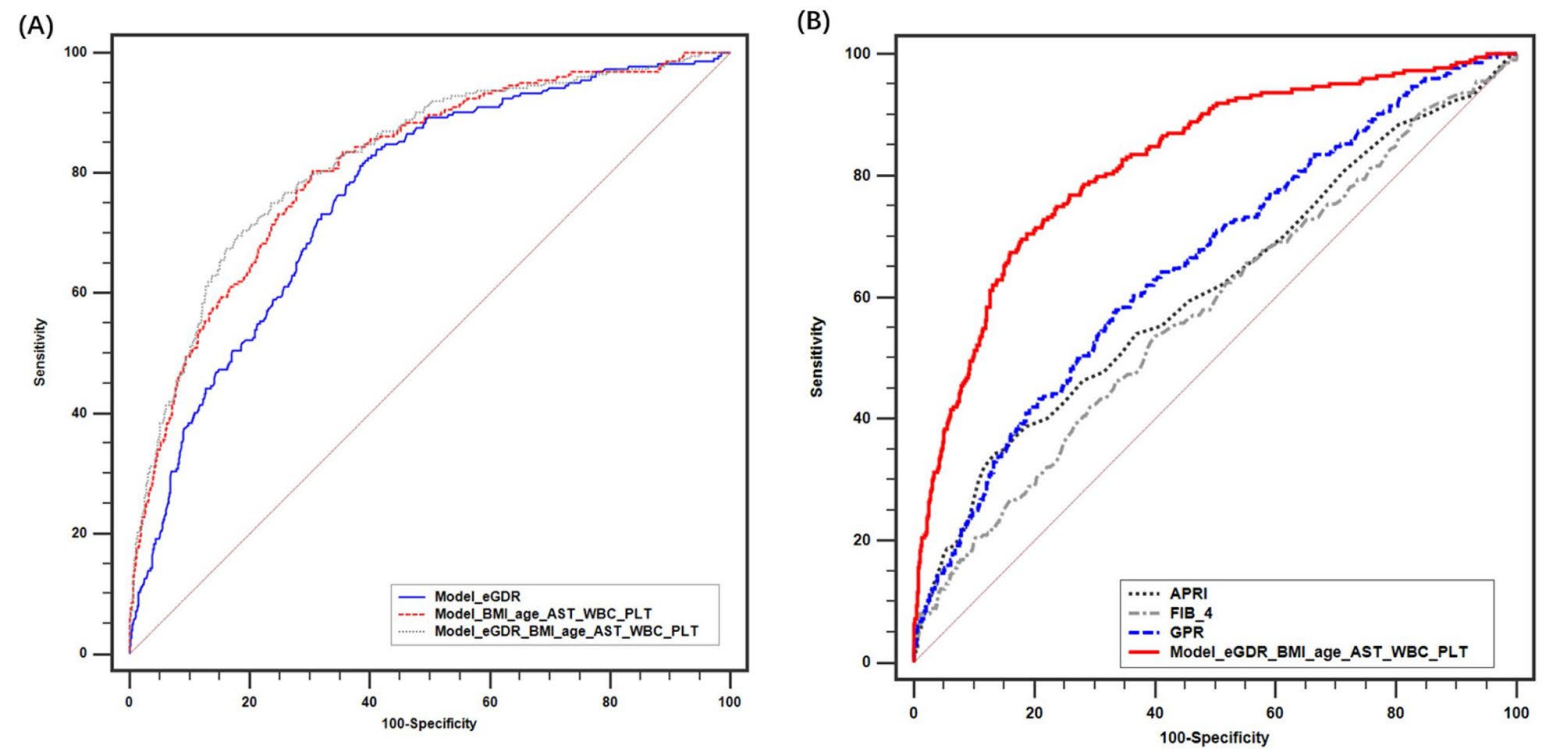
Comparison between Model(eGDR_BMI_age_AST_WBC_PLT), Model(eGDR), and Model(BMI_age_AST_WBC_PLT) (**A**), and between APRI, FIB-4, and GPR (**B**). ALT, Alanine aminotransferase; APRI, aminotransferase-to-platelet ratio index; AST, aspartate transaminase; BMI, body mass index; eGDR, estimated glucose disposal rate, FIB-4, fibrosis-4; GGT, γ-glutamyl transferase; NAFLD, nonalcoholic fatty liver disease; PLT, platelet; WBC, white blood cell

The AUROC of Model(eGDR_BMI_age_AST_WBC_PLT) was 0.822, which was significantly higher than that of Model(eGDR) (AUROC = 0.763, *P* < 0.001) and Model (BMI_age_AST_WBC_PLT) (AUROC = 0.811, and *P* = 0.02) (Fig. 3A). Moreover, the AUROC of Model(eGDR_BMI_age_AST_WBC_PLT) was also significantly higher than that of APRI, GPR, and FIB-4 (AUROC = 0.613, 0.657, and 0.579, respectively, all *P* < 0.001) (Fig. 3B). With an optimal cutoff value of 0.179, Model(eGDR_BMI_age_AST_WBC_PLT) achieved 69.64% sensitivity and 82.29% specificity (Youden index=0.519).

### Application of the new model in participants with obesity, overweight, and normal weight

**Fig. 4:**
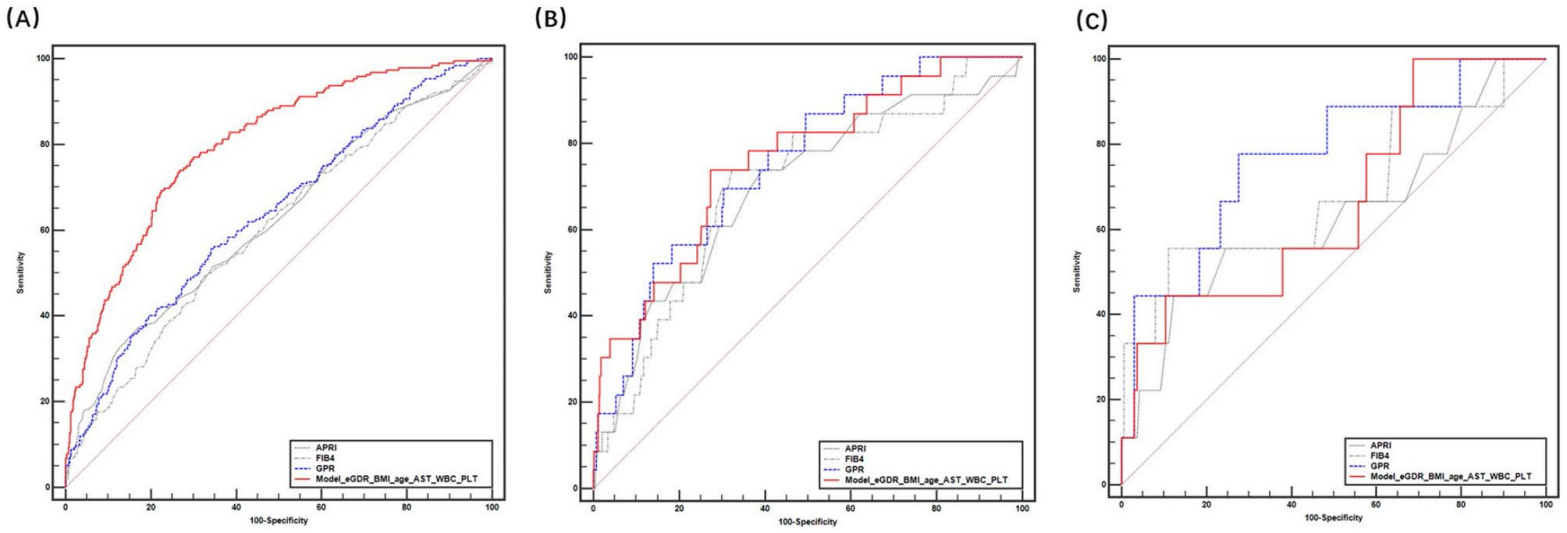
Application of Model(eGDR_BMI_age_AST_WBC_PLT) for evaluating fibrosis in individuals with NAFLD and obesity (**A**), overweight (**B**), and normal weight (**C**). AST, Aspartate transaminase; BMI, body mass index; eGDR, estimated glucose disposal rate; NAFLD, nonalcoholic fatty liver disease; PLT, platelet; WBC, white blood cell

For patients with NAFLD and obesity, the AUROCs of APRI, FIB-4, and GPR in predicting significant fibrosis were 0.623, 0.603, and 0.637, respectively. However, the AUROC of the Model(eGDR_BMI_age_AST_WBC_PLT) was 0.798, which was significantly higher than those of APRI, FIB-4, and GPR (all *P* < 0.001). Moreover, no significant difference was observed among the participants with NAFLD and overweight or normal weight (all *P* > 0.05) **(**Fig. 4**)**.

## Discussion

The present study investigated the role of eGDR in predicting significant fibrosis. The data from 1585 individuals with NAFLD showed that eGDR negatively correlated with fibrosis degrees was an independent risk factor for liver fibrosis. A new model incorporating eGDR, BMI, age, AST, WBC, and PLT had higher accuracy than conventional NSSs for individuals with NAFLD and obesity.

Increasing evidence shows an important role of IR in NAFLD development and fibrosis progression [12, 13]. Thus, we hypothesize that eGDR, which is an IR-related index, may be associated with liver fibrosis. The data from participants with NAFLD and obesity showed a negative correlation of eGDR with liver fibrosis, and a positive correlation of CRP and WBC with liver fibrosis. In addition, eGDR demonstrates a negative correlation with both metabolic disturbance indices (CAP, BMI, and WC) and nonspecific inflammatory markers (CRP levels and WBC counts) in overweight and obese individuals. In contrast, among normal-weight individuals, eGDR shows negative correlations exclusively with metabolic parameters (CAP, BMI, WC, TC, and TG levels), but not with inflammatory markers. Hypersensitive CRP level has been reported to be associated with NAFLD in nonobese individuals [23]. Given that non-obese individuals with varying degrees of fibrosis severity exhibit significant alternations in microbiome diversity [24], gut microbiome-induced nonspecific inflammation may play a more prominent role than metabolic disorders in the progression of liver fibrosis. It is unlikely that IR alone can fully explain the relationship between eGDR and liver fibrosis, thus warranting further investigation. These findings suggest a potential interaction between nonspecific inflammation, gut microbiome and IR, all of which may contribute to the pathogenesis of fibrosis progression. Previous studies have revealed that gut microbial therapy including probiotics and synbiotics, could significantly reduce serum CRP levels among NAFLD patients [25]. IR, lipid indices, liver steatosis and fibrosis could also be improved by microbiome-targeted therapies [26–28]. The dynamics of eGDR during microbiome-targeted therapies may represent a promising area for future research.

Obesity coupled with NAFLD is common in clinical practice and presents as a complex IR-related issue. Adults with significant liver fibrosis should be considered for pharmacological intervention [29]. Although BMI, age, AST, WBC, and PLT are independent risk factors for significant fibrosis, eGDR can increase the predictive accuracy of these parameters. The new model incorporating eGDR, BMI, age, AST, WBC, and PLT was superior to conventional NSSs for individuals with NAFLD and obesity. Thus, it is a useful and convenient method for significant fibrosis surveillance. We should pay more attention to waist circumference, hypertension, and HbA1c, especially in patients with obesity.

This study had certain limitations. First, it is a cross-sectional study without long-term follow-up. Hence, the dynamic changes in eGDR and liver fibrosis over a long time should be compared. Second, we evaluated liver fibrosis using the noninvasive FibroScan model 502 V2 Touch, which introduced potential selection bias, including diagnostic bias. The clinical data from liver biopsies are needed to validate these findings. Liver biopsy, being invasive and inconvenient for monitoring fibrosis progression during follow-up, has seen limited widespread use in clinical practice due to these constraints. FibroScan, offering valuable insights into fibrosis severity and prognosis, has emerged as an alternative method for managing liver fibrosis. However, the accuracy of FibroScan can be compromised by factors such as liver inflammation, severe obesity, significant ascites, congestion, or cholestasis [30]. Moreover, FibroScan is not available in many hospitals or regions, highlighting the need for optimal non-invasive assessment techniques. Third, the mechanism underlying the nonsignificant correlation of eGDR with liver fibrosis in “lean NAFLD” remains unclear.

Furthermore, emerging evidence suggests that a rapid ascending trajectory of BMI demonstrates a significant association with advanced fibrosis [31]. Given that NAFLD represents a multifactorial disease entity, additional determinants, such as socioeconomic status, dietary patterns, physical activity, medication regimens, socioeconomic status, and hepatic enzyme profiles beyond conventional ALT and AST measurements, as well as longitudinal changes in both BMI and waist circumference, may substantially influence both eGDR and fibrosis progression. Although heavy drinks were excluded from the analysis based on previous publications, the potential overlap between alcohol-related liver damage and NAFLD cannot be entirely ruled out. These compelling findings underscore the necessity for well-designed cohort studies to systematically evaluate the impact of these potential confounding factors on hepatic fibrogenesis.

## Conclusions

Low eGDR is negatively correlated with liver fibrosis in individuals with NAFLD and obesity. A model that includes eGDR, BMI, age, AST levels, WBC count, and PLT count demonstrates strong predictive value for evaluating fibrosis.

## Data Availability

No datasets were generated or analysed during the current study.

## Abbreviations

ALT: Alanine aminotransferase
AST: Aspartate transaminase
BMI: Body mass index
CAP: Controlled attenuated parameter
CRP: C-reactive protein
eGDR: Estimated glucose disposal rate
GGT: γ-glutamyl transferase
NAFLD: Nonalcoholic fatty liver disease
PLT: Platelet
TC: Total cholesterol
TG: Triglyceride
UA: Uric acid
WBC: White blood cell
NSSs: Noninvasive scoring systems
APRI: Aminotransferase-to-platelet ratio index
FIB-4: Fibrosis-4
GPR: Gamma-glutamyl transpeptidase to platelet ratio
WC: Waist circumference
HbA1c: Hemoglobin A1c
